# *LIGHT/BTLA* polymorphisms and antibody-mediated-rejection after heart transplantation

**DOI:** 10.18632/oncotarget.26327

**Published:** 2018-11-09

**Authors:** Grecia M. Marrón-Liñares, Lucía Núñez, Manuel Hermida-Prieto

**Affiliations:** Instituto de Investigación Biomédica de la Universidad de A Coruña (INIBIC), Complexo Hospitalario Universitario de A Coruña (CHUAC)-Universidad de A Coruña, A Coruña, Spain

**Keywords:** AMR, heart transplant, LIGHT/BTLA genes

Antibody-mediated rejection (AMR) is one of the main problems after transplantation, partly due to the unclear aspect of the pathogenesis of AMR. Allograft rejection caused by antibodies can be mediated by different mechanisms. Classically, antibodies, produced by B-cells, induce acute rejection through the fixation and activation of the complement cascade, resulting in tissue injury [1]. Searching for new approaches to understand the pathogenesis of AMR, HVEM/LIGHT/BTLA/CD160 coestimulatory/coinhibitory signaling pathway has emerged as a potential target for immune therapy after transplantation [2].

There have been many attempts to associate solid organ allograft outcomes with specific genetic variants [3]. In fact, our group has recently found that polymorphisms in recipients and donors in genes of the complement and B-cell pathways [4-5] have been associated with AMR. However, the study of gene polymorphisms in other pathways, like HVEM/LIGHT/BTLA/CD160, could be interesting due to its role in costimulation/coinhibition of T and B cell responses, enhancing immune responses. In fact, in a recent article, published in the Vol.8 of *Oncotarget,* Wang *et al.* [6] explore the association between *HVEM/LIGHT/BTLA/CD160* polymorphisms and AMR after kidney transplantation. The authors used next generation sequencing (NGS) to evaluate the association between 41 single nucleotide polymorphisms (SNPs) of *HVEM/LIGHT/BTLA/CD160* genes in 200 renal recipients, 69 with AMR and 131 that were considered stable. Wang *et al* did not found any significant association between these polymorphisms and AMR in renal transplantation.

Based on the study of Wang *et al,* and in view of the relevant role of HVEM/LIGHT/BTLA/CD160 pathway in the immune response, we retrospectively analyzed the presence of SNPs in *LIGHT/BTLA* genes by NGS, due to their importance as HVEM ligands. For this purpose, 46 heart transplant recipients were analyzed, 23 with AMR and 23 matched controls without AMR. In this study, we have identified 7 SNPs in *LIGHT/BTLA* genes (Table 1) but, after statistical analysis, no significant association between these SNPs and AMR in heart transplantation was found.

**Table 1:** Genotype distribution of LIGHT/BTLA polymorphisms in patients with AMR and controls after heart transplantation

Gene	Genotype	Position	AMR (*n*=23)	Control (*n*=23)	*P*-value
***LIGHT***	rs344560 T/T T/C C/C	Chr19:6665020	0 5 18	1 6 16	0.48
	rs377184644 G/G G/A	Chr19:6665196	22 1	23 0	1.00
	rs143257425 G/G G/A	Chr19:6665198	23 0	22 1	1.00
	rs2291668 G/G G/A	Chr19:6669934	15 8	17 6	0.72
***BTLA***	rs9288952 G/G G/A A/A	Chr3:112185025	0 3 20	2 3 18	0.48
	rs76844316 T/T T/G	Chr3:112188609	23 0	22 1	1.00
	rs16859633 T/T T/C	Chr3:112198335	23 0	22 1	1.00

Despite the negative results obtained by both Wang *et al.* and our research group in renal and cardiac transplant respectively, there is strong evidence suggesting the involvement of this pathway in immune regulation and graft rejection [2, 7]. A possible hypothesis for this is that HVEM/LIGHT/BTLA/CD160 pathway could be more related with cellular rejection (CR) than with AMR, due to the relevant role of this pathway in the regulation of T cells [2]. However, more research must be done to verify the role of *HVEM/LIGHT/BTLA/CD160* polymorphisms in CR, without ruling out their possible role in AMR. It should be recalled that these genes are expressed in other cell types like B cells, NK cells, DCs, and macrophages, which are involved in AMR [1, 7]. Therefore, further research should be done in larger cohorts in order to elucidate the role of *HVEM/LIGHT/BTLA/CD160* polymorphisms in the progression of AMR and CR.

## References

[1] Colvin RB (2005). Nat Rev Immunol.

[2] del Río ML (2010). J Leukoc Biol.

[3] Günesacar R (2015). Tissue Antigens.

[4] Marrón-Liñares GM (2018). J Heart Lung Transplant.

[5] Marrón-Liñares GM (2018). Circ J.

[6] Wang Z (2017). Oncotarget.

[7] Steingberg M (2011). Immunol Rev.

